# Monitoring non-small cell lung cancer progression and treatment response through hyaluronic acid in sputum

**DOI:** 10.1590/1414-431X2021e11513

**Published:** 2022-01-25

**Authors:** J. Chinoca, D.S. Andrade, A. Mendes, P. De Marchi, T.G. Prieto, C.M. Baldavira, C. Farhat, J.R.M. Martins, H.B. Nader, D.M. Carraro, V.L. Capelozzi, V. de Sá

**Affiliations:** 1 Laboratório de Genômica e Biologia Molecular, AC Camargo Cancer Center, São Paulo, SP, Brasil; 2 Departamento de Patologia, Faculdade de Medicina, Universidade de São Paulo, São Paulo, SP, Brasil; 3 Departamento de Oncologia Médica, Hospital de Amor de Barretos, São Paulo, SP, Brasil; 4 Departamento de Bioquímica, Disciplina de Biologia Molecular, Escola Paulista de Medicina, Universidade Federal de São Paulo, São Paulo, SP, Brasil; 5 Laboratório de Endocrinologia Molecular e Translacional, Escola Paulista de Medicina, Universidade Federal de São Paulo, São Paulo, SP, Brasil; 6 Departamento de Bioquímica, Instituto de Química, Universidade de São Paulo, São Paulo, SP, Brasil

**Keywords:** Sputum, Lung cancer, Hyaluronan, Radiologic response, Treatment and outcome

## Abstract

We evaluated whether hyaluronan (HA) levels in the sputum could be used as a noninvasive tool to predict progressive disease and treatment response, as detected in a computed tomography scan in non-small cell lung cancer (NSCLC) patients. Sputum samples were collected from 84 patients with histological confirmation of NSCLC, 33 of which were in early-stage and 51 in advanced-stage disease. Patients received systemic chemotherapy (CT) after surgery (n=36), combined CT and immunotherapy (IO) (n=15), or targeted therapy for driver mutation and disease relapse (N=4). The primary end-point was to compare sputum HA levels in two different concentrations of hypertonic saline solution with overall survival (OS) and the secondary and exploratory end-points were radiologic responses to treatment and patient outcome. Higher concentrations of HA in the sputum were significantly associated to factors related to tumor stage, phenotype, response to treatment, and outcome. In the early stage, patients with lower sputum HA levels before treatment achieved a complete tumor response after systemic CT with better progression-free survival (PFS) than those with high HA levels. We also examined the importance of the sputum HA concentration and tumor response in the 51 patients who developed metastatic disease and received CT+IO. Patients with low levels of sputum HA showed a complete tumor response in the computed tomography scan and stable disease after CT+IO treatment, as well as a better PFS than those receiving CT alone. HA levels in sputum of NSCLC patients may serve as a candidate biomarker to detect progressive disease and monitor treatment response in computed tomography scans.

## Introduction

In recent years, innovative-targeted drug therapies have emerged as a result of our deeper understanding of the molecular biology of malignant tumors. Although these therapies have improved the outcome of non-small cell lung cancer (NSCLC) patients (1), lung cancer remains the world’s deadliest type of cancer to this day (2). This increased mortality in lung cancer patients is often associated with three main causes: the absence of an established screening protocol to detect early-stage disease, the fact that the first symptoms usually arise in advanced stages, and the lack of biomarkers to monitor therapeutic response and to detect disease progression and poor prognosis (3). Therefore, there is a pressing need to explore non-conventional and non-invasive procedures to identify new biomarkers that can help improve patient outcome.

The genomic, proteomic, and transcriptomic profiling of lung cancers is now being complemented by the study of the biochemical properties of endogenous metabolites produced by malignant cells (4). In this regard, a major polysaccharide component of the extracellular matrix, hyaluronan (HA), has attracted the attention of researchers because of its biochemical properties and its ability to control cell proliferation and migration through interactions with cell-surface receptors and binding molecules (5). HA is primarily considered an extracellular molecule, but it can also be found inside cells around the perinuclear area during mitosis and in cytoplasm organelles (6).

In the respiratory system, some studies show that HA and its degradation products play a key role in the physiopathology of chronic obstructive pulmonary diseases (7). Lung injuries induce the release of short-fragment HA, which in turn activates innate immune receptors, often resulting in inflammation, remodeling, and hyperresponsiveness, in addition to other clinical symptoms. Increased HA levels have also been associated with poor prognosis and survival in malignant cancers, including in lung (8), pleura (9), and breast (10) tumors. Together, these findings underline the importance of both HA biosynthesis and degradation to detect extracellular and cellular injury and demonstrate the need for novel techniques that allow us to predict response and the probability of disease progression. Functional imaging modalities based on computed tomography scan, magnetic resonance imaging, and ultrasound are promising noninvasive ways to determine changes in tumor growth (11).

Given the above scenario, we hypothesized that HA levels may be a powerful candidate for an *in situ* non-invasive diagnostic tool that can provide physicians with a specific metabolic phenotype of NSCLC, obtained from sputum, to detect progressive lung disease and monitor treatment. In summary, we divided patients into two distinct populations according to their sputum HA levels, and assessed their computed tomography scans to investigate the potential of HA to predict treatment response. The resulting data led to different prognostic implications in early and advanced stages and raised the possibility that HA levels in the sputum of NSCLC patients may reflect the biochemical reprogramming of endogenous machinery in malignant cells during progressive disease.

## Material and Methods

### Patients

Patients were recruited at the AC Camargo Cancer Center and the Hospital de Amor de Barretos and signed an informed consent upon entry. The study protocol, compliant to the ethical guidelines of the Declaration of Helsinki, was approved by the Ethics Committees of both participating institutions (process number 2237/16). Altogether, we collected sputum samples from 84 patients with histological confirmation of NSCLC, including surgically resected specimens (n=36) and biopsies (n=19) from AC Camargo Cancer Center and from Hospital de Amor de Barretos (n=29). The clinicopathological data collected included gender, age, tobacco history, histology, and disease stage, as described in the Eighth Edition of the Union for International Cancer Control (UICC) TNM Classification of Malignant Tumors (12). We also obtained information regarding systemic or locoregional treatments, eventual disease relapse, and death. Patients were followed-up through monthly visits to the oncologist. Brain, chest, and abdomen CT scans were performed every 6 months for the first 2 years and every year thereafter. Overall survival (OS) was the primary end-point and was defined as the interval from surgery to death or last contact, whereas the main secondary and exploratory end-points of the study were radiologic response to treatment and patient outcome.

### Sputum collection

Sputum induction was first performed by the inhalation of two different concentrations of hypertonic saline solution (3 and 7%) for efficiency comparison, using an ultrasonic nebulizer (2000 Ultra-neb 2000, Devilbiss, Sunrise Medical, USA) for 7 min, with a maximum of 3 inhalations. The sputum samples were then separated from saliva by centrifugation (800 *g* at 4°C for 10 min) and stored at -80°C until determination of the HA concentration.

### Hyaluronic enzyme-linked immunosorbent assay (HA-ELISA)

Sputum samples were thawed and incubated with 7 M urea at 60°C until the complete breakdown of the sputum’s hydrophobic associations. HA was determined by a noncompetitive fluoroassay developed in our laboratory (13). Briefly, we coated the ELISA plate by adding 100 µL of HA-binding protein at a concentration of 1 mg/mL (PL) diluted in carbonate buffer (pH 9.6/0.06 M) at 4°C for 8 h. After coating, we blocked spaces not filled by PL in the previous step by adding 200 µL of 1% albumin diluted in 1% TRIS/BSA buffer to each well and then incubated the plates for 4 h at room temperature. The ELISA plates were washed 3 times with wash solution (0.05 M Tris-HCl, pH 7.75) and decanted after the last wash. The protein concentration in each sputum sample was measured by a BCA Protein assay (Thermo Scientific, USA), a detergent-compatible formulation based on bicinchoninic acid (BCA) used for the colorimetric detection and quantitation of total protein. Sputum HA concentration was measured after proteolysis with 4 mg/mL Maxatase (Biocon do Brasil Industrial, Brazil), pH 8.0, 0.15 M NaCl, 50 mM Tris-HCl) by overnight incubation at 60°C. Standard HA solutions (0-1000 ng/mL) and patient samples were diluted in working buffer (0.05 M Tris-HCl, pH 7.75 + 1% bovine albumin), placed in triplicate in ELISA plates previously coated with the HA-binding protein, and left to rest for 8 h at 4°C. Next, the plate was incubated with the biotin-labeled HA-binding protein for 2 h. After 3 washes with washing buffer (0.05 M Tris-HCl, pH 7.75), the plate was incubated with europium-labeled streptavidin for 30 min at room temperature. After washing the plate with washing buffer, an enhancement solution (Wallac Oy, Sweden) was added, and the plate was shaken gently for 10 min at room temperature. Finally, the plate was placed in a fluorimeter (Victor 2, Wallac Oy) to determine the emitted fluorescence. A calculation program (MultiCalc software program, Perkin-Elmer Life Sciences - Wallac Oy, Sweden) was used to interpret the data (counts/s) and provide the results of the HA concentration in ng/mg protein.

### Statistical analysis

We used either a chi-squared test or Fisher’s exact test to compare categorical variables, and the Wilcoxon rank-sum test and Kruskal-Wallis test to detect differences in continuous variables between groups of patients. We also applied the general regression linear model to determine the association between continuous variables and several other variables and examined the residuals to ensure that they were approximately normally distributed. A unidirectional variance analysis was performed to compare the HA concentration in the sputum among lung cancer patients, cancer-free patients, and healthy volunteers. OS curves were estimated using the Kaplan-Meier method, with OS being defined as the interval from surgical resection, combined with chemotherapy (CT) or chemotherapy + immunotherapy (CT+IO), to death. The difference in survival times between distinct groups of interest was assessed using the log-rank test, whereas the OS regression analysis was performed using the Cox proportional hazards model. Variables shown to be significantly associated with survival by the univariate analysis were then entered in a multivariate Cox proportional hazards regression model. Also, receiver operating characteristic (ROC) curves were drawn to determine the cut-off of sputum HA concentration that yielded the best possible differentiation among cancer patients, cancer-free individuals, and healthy volunteers. These analyses were performed using two statistical packages: IBM SPSS (version 22; USA) and S-Plus (version 8.04; TIBCO, USA). All data with a P-value ≤0.05 were deemed statistically significant.

## Results

**Figure 1:**
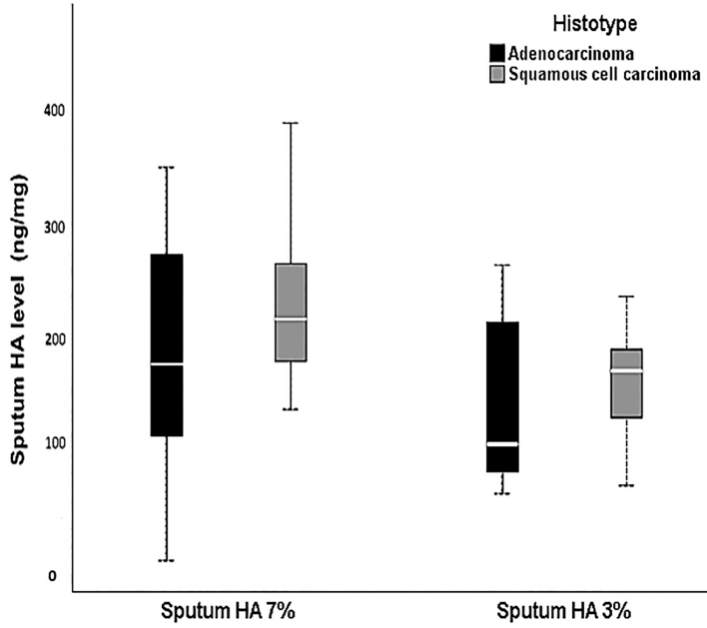
Plots demonstrate the differences between sputum hyaluronan (HA) levels collected with 7% NaCl or with 3% NaCl at the time of diagnosis, stratified by adenocarcinoma and squamous cell carcinoma histotypes. Solid bars represent HA levels in ng/mg between the 25th and 75th percentiles, the white bar shows the median value, and the top and bottom brackets show the extreme values. The Wilcoxon rank-sum test was used for analyses.

The clinical characteristics of the patients in our cohort are summarized in Table 1 and stratified according to the concentration of saline used to collect the sputum. Male patients older than 58 years had a significantly higher HA concentration in sputum samples collected with 7% saline (P=0.01), as did smokers (P=0.002) and patients with squamous cell carcinoma (P=0.03). HA concentrations in sputum collected with 7% saline were marginally different according to cancer stage (P=0.06). Interestingly, the higher HA concentration found in samples collected with 7% saline was significantly associated with progressive disease after systemic treatment, as assessed by computed tomography scan (P=0.01). In *KRAS*-mutant tumors, the HA concentration in sputum collected with 7% saline also tended to be higher than in those collected with 3% saline (P=0.05). Tumor type also seemed to impact HA concentrations. Patients with central lesions, such as squamous cell carcinomas involving the tracheobronchial tree, had significantly higher sputum HA levels compared to those with more peripheral disease, such as a peripheral adenocarcinoma (Figure 1).

**Table 1:** Clinicopathological characteristics of the cancer patients stratified according to sputum hyaluronic acid (HA) level.

Patient characteristics	HA concentration (ng/mg) in 7% saline	HA concentration (ng/mg) in 3% saline	P-value
Age (years)			0.01
≤58	190.87	33.26	
>58	192.12	122.47	
Gender			0.001
Female	157.14	15.19	
Male	215.48	108.73	
Tobacco history			0.002
Non-smokers	187.31	18.28	
Former smokers	169.03	46.05	
Smokers	206.41	93.41	
Tobacco level			0.08
2 packs/year	185.63	39.60	
60 packs/year	199.37	90.96	
Histologic types			0.03
Adenocarcinoma	159.41	42.62	
Squamous cell carcinoma	432.54	93.89	
IASLC clinical stage^†^			0.06
I	119.17	0.00	
II	81.28	11.40	
III	275.76	39.27	
Tumor stage^†^			0.05
T1	83.50	14.95	
T2	187.01	42.90	
T3	281.14	145.28	
T4	202.32	43.95	
Lymph node status^†^			0.16
N0	174.21	133.39	
N1	242.94	34.00	
N2	193.43	53.46	
Tumor response (CT-scan)			0.01
Partial response	118.97		
Complete response	211.00		
Stable disease	117.38	81.68	
Progressive disease	292.78	61.30	
Mutational status			0.05
Wild-type	240.13	75.57	
ALK	219.00	2.62	
EGFR	34.11		
KRAS	228.00		
RET	15.89		
EGRF + T790M	122.60		
Systemic treatment			0.01
CT	146.65		
CT + IO	101.11		

Univariate general linear model controlled for HA level, HA in 7 and 3% saline, and characteristics of the patients. ^†^7th International Association for the Study of Lung Cancer (reference 12). CT-scan: computed tomography scan; CT: chemotherapy; IO: immunotherapy. Data are reported as mean. The General Linear Model test was used for statistical analyses.

**Figure 2:**
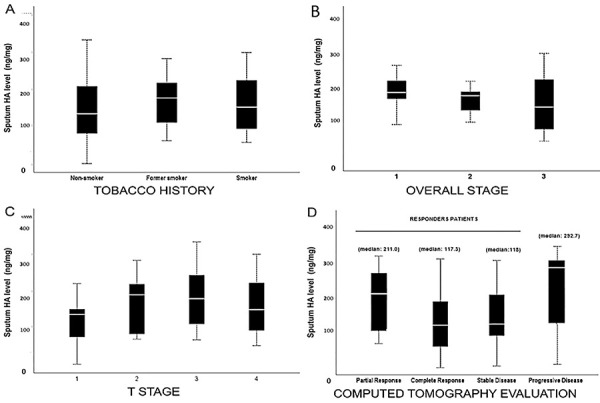
Relationships between sputum hyaluronan (HA, ng/mg, 7% NaCl) level and tobacco history (**A**), overall stage (**B**), T stage (**C**), and tumor response evaluation by computed tomography scans, after systemic treatment, classified as partial response, complete response, stable disease (responder patients), or progressive disease, according to the response evaluation criteria in solid tumors (RECIST) (**D**). The solid bar represents the values of HA between the 25th and 75th percentiles, the white line indicates the median value, and the top and bottom brackets show the extreme values. Fisher’s exact test was used for analyses.

Figure 2 shows the associations between the sputum HA concentration collected with 7% saline and tobacco history (panel A), pathological stage (panel B), T stage (panel C), and the tumor response evaluation (panel D) by computed tomography scans after systemic treatment, classified as partial response, complete response, stable disease (responder patients), or progressive disease, according to the response evaluation criteria in solid tumors (RECIST). The median HA levels in complete response (median=117.3 ng/mg) and stable disease (118 ng/mg) were significantly lower than HA levels in patients with partial response (211 ng/mg) and disease progression (292.7 ng/mg) (P=0.02).

### Survival analysis

Overall, early NSCLC disease [stages I (n=6), II (n=5), and IIIA (n=22)] was detected in 33 (39%) patients, whereas advanced disease (stage IV) was found in 51 (61%) patients. Of the 51 patients from the AC Camargo Cancer Center, 36 (70%) underwent surgery for early NSCLC disease followed with systemic CT (cisplatin + pemetrexed), 4 (8%) with gefitinib for driver mutation, and 11 (22%) received CT+IO (carboplatin + pemetrexed + pembrolizumab) for advanced NSCLC disease. Of the 84 patients, 30 (35.7%) died due to disease progression. The median follow-up was 9 months (range, 3 to 71 months).

**Figure 3:**
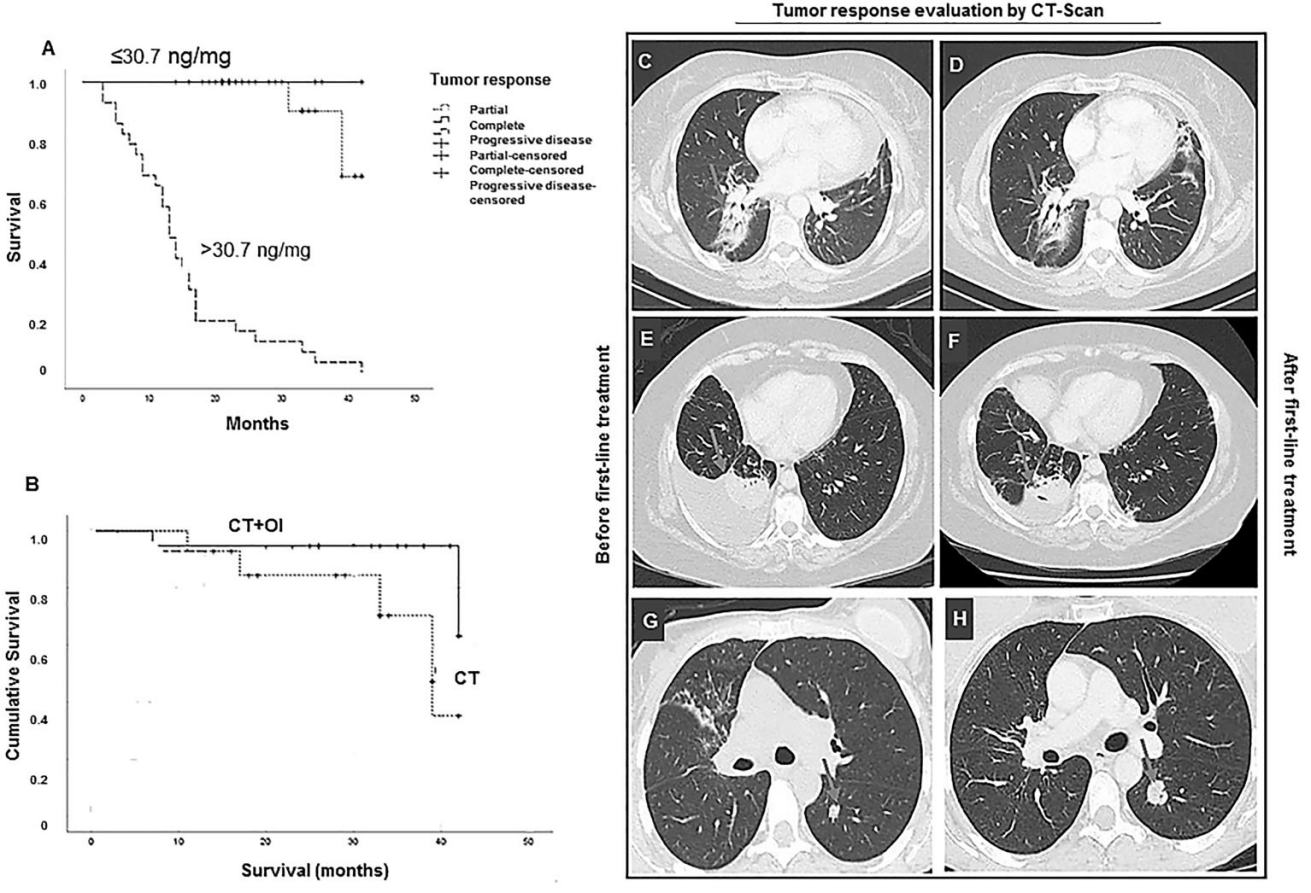
Kaplan-Meier plots of survival probability *vs* follow-up time in months in patients with tumor response evaluation confirmed by computed tomography scan (CT-scan), after first line-therapy. **A**, The group with hyaluronan (HA) ≤30.7 ng/mg in 7% NaCl in sputum appears as the top curve and the group with HA >30.7 ng/mg in sputum appears as the bottom curve. **B**, The group of patients treated with systemic chemotherapy + immunotherapy (CT+IO) appears as the top curve and the group treated only with chemotherapy (CT) appears as the bottom curve. **C**-**H,** The images show the tumor response evaluation by CT-scan after first-line therapy. **C** and **D**, Patient with stable disease, **E** and **F** patient with partial response, and **G** and **H**, patient with progressive disease.

In early NSCLC disease, patients with pathological stage I or II showed very similar survival outcomes, with a median survival time of 30 months in both groups and were thus grouped together. The results of the Cox regression analysis in early NSCLC disease are shown in Table 2. A univariate Cox analysis demonstrated that tumor stage, stages I and II, HA ≤30.70 ng/mg, and computed tomography scan tumor response were significantly related to low risk of death and better survival. When these variables were introduced in a multivariate Cox analysis, patients with sputum HA ≤30.70 ng/mg before systemic treatment showed a greater tumor response and lower risk of death compared to those with sputum HA >30.70 ng/mg. Tumor response was assessed by a computed tomography scan after treatment in different patient groups and their median survival times are plotted in Figure 3A. The groups that had complete response and stable disease are shown in the top curve. Although their median survival time was not reached during follow-up, their mean survival time was quite long (30 months).

**Table 2:** Univariate and multivariate analysis using a Cox proportional hazards model of the variables associated with overall survival (OS).

Variables	Univariate analysis			Multivariate analysis		
	Chi-squared	β coefficient	P-value	β coefficient	Chi-squared	P-value
Age (years)	0.540		0.45			
≤58		-0.270				
>58 (reference)		1				
Gender	0.748		0.386			
Female		-0.336				
Male (reference)		1				
Tobacco history			0.744			
Non-smoker	0.599	0.130				
Former smoker		-0.287				
Smoker (reference)		1				
Tobacco level	0.359		0.549			
2 packs/year		0.221				
60 packs/year		1				
Histologic types	3.724		0.155			
Adenocarcinoma		-0.349				
Squamous cell carcinoma (reference)		1				
IASLC stage	11.962		0.01		17.53	0.014
I+II		-13.479		-8.350		
III (reference)		1		1		
Tumor stage	9.133		0.02		17.50	0.014
T1		-1.825		-0.816		
T2		-0.806		0.725		
T3		-0.311		0.682		
T4 (reference)		1		1		
Lymph node status	1.156		0.76			
N0		-0.349				
N1		0.212				
N2 (reference)		1				
Mutation status	6.740		0.24			
Wild type		10.454				
ALK		10.439				
EGFR		9.570				
KRAS		9.941				
RET		0.178				
EGFR + T790M (reference)		1				
Pretreatment HA level (ng/µg)	0.472		0.05		93.33	0.0001
≤30.70		-0.299		0.623		
>30.70 (reference)		1				
Computed tomography post treatment	78.853		0.0001		93.34	0.0001
Partial response		-13.583		18.196		
Complete response		-2.981		1.380		
Stable disease		-13.550		21.345		
Progressive disease (reference)		1		1		
Systemic treatment						
CT	14.665	1.746	0.013	1.334	24.846	0.001
CT+IO (reference)		1		1		

CT: chemotherapy; CT+IO: chemotherapy + immunotherapy.

We also examined the impact of sputum HA concentration and tumor response on the risk of death in the 51 patients with advanced NSCLC disease who received CT+IO. In this scenario, patients with sputum HA ≤30.70 ng/mg before systemic treatment presented a complete tumor computed tomography scan response and stable disease after CT+IO treatment with a better progression-free survival than those receiving only CT (P=0.01; Table 3). There were differences in the median survival times of patients treated with CT or CT+IO, which are illustrated by the Kaplan-Meier plots shown in Figure 3B. The group treated with CT+IO (top curve) had a median survival time of 40.1 months, whereas those treated with CT only (bottom curve) had a median survival time of 17.7 months (P=0.03; log-rank test).

**Table 3:** Cox proportional hazard model of survival time in a cohort of 51 patients with metastatic disease.

Variable	Coefficient	Standard error	P-value
CT-scan tumor response	0.543	0.207	0.001
CT + IO	0.448	0.190	0.01
HA (ng/mg) in 7% saline	0.130	0.055	0.02

CT-scan: computed tomography scan; CT+IO: chemotherapy + immunotherapy; HA: hyaluronan.

### Diagnostic power of HA sputum level at 3% and 7% NaCl

**Figure 4:**
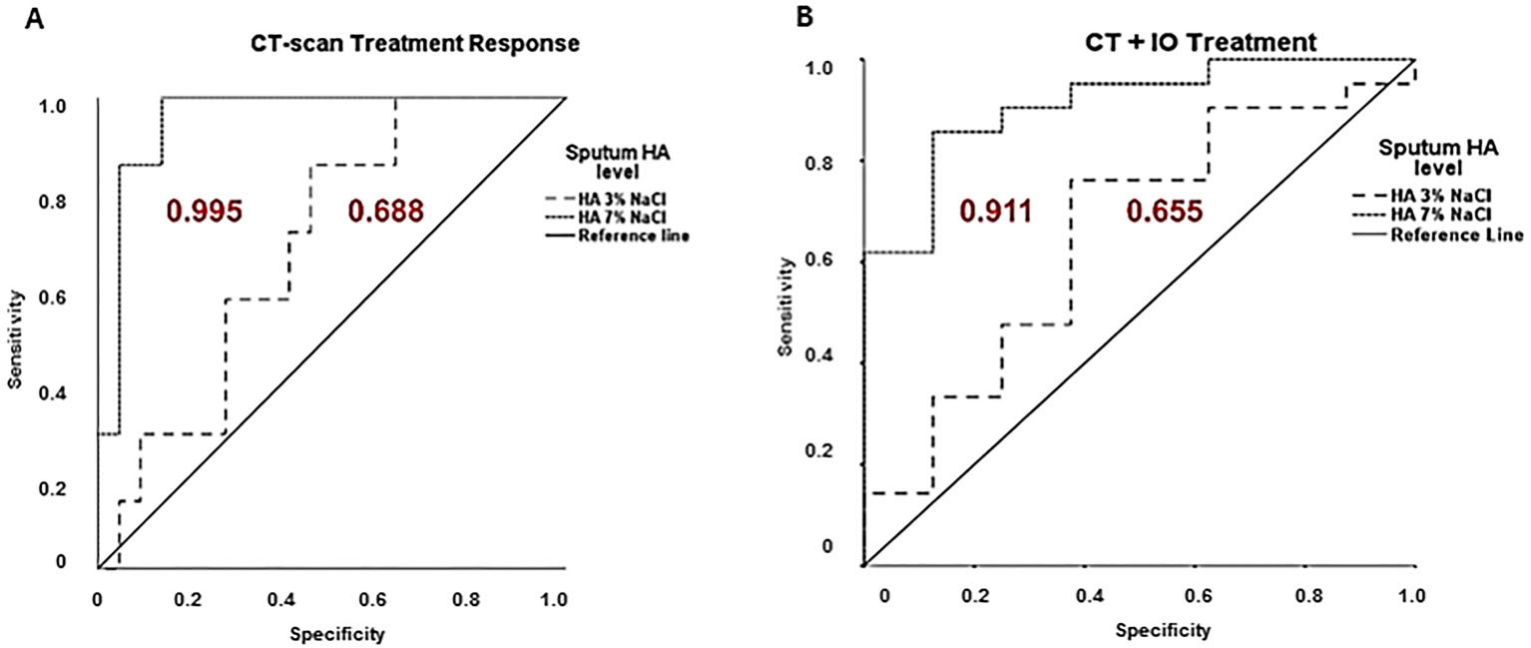
Receiver operating characteristic curves for sputum levels of hyaluronan (HA). **A**, The cut-off level of HA in 7% NaCl that resulted in the highest diagnostic accuracy was 73.30 ng/mg compared to HA in 3% NaCl. This cut-off point discriminated between complete response on computed tomography scan (CT-scan) and progressive disease, with 100% sensitivity and 86% specificity. The diagnostic accuracy was 95%. **B**, The cut-off level of HA in 7% NaCl that resulted in the highest diagnostic accuracy was 44.70 ng/mg. This cut-off point discriminated between CT and CT+IO treatment, with 85% sensitivity and 87% of specificity. The diagnostic accuracy was 91%.

We used a ROC curve to analyze the extent to which a sputum HA analysis could help predict tumor response, as well as treatment response. When assessing complete response and progressive disease, the area under the curve for sputum HA 7% saline was 0.995 (0.881-1.000; P=0.0001) whereas that of HA 3% saline was 0.688 (0.487-0.890; P=0.139). Assuming a cut-off value of 77.30 ng/mg for HA 7% saline, the specificity was 86% and the sensitivity was 100% (Figure 4A). Alternatively, when used to assess response to CT and CT+IO, the area under the curve for sputum HA 7% saline was 0.911 (0.800-1.000; P=0.01), whereas that for HA 3% saline was 0.655 (0.424-0.885; P=0.205). Assuming a cut-off value of 44.70 ng/mg for HA 7% saline, the specificity was 87% and the sensitivity was 85% in patients undergoing CT, and 85% sensitivity and 87% specificity in CT+IO treatments. The diagnostic accuracy was 91% (Figure 4B).

## Discussion

By examining the clinicopathological characteristics of our cohort of patients, we advocate that the likely reason for surgery resection failing to cure certain patients with early-stage NSCLC is because progressive disease often goes undetected by routine pathological analysis. In fact, of the 84 patients in our cohort, 35.7% died due to disease progression. Therefore, the question of interest is whether additional technological and biological information collected from either the malignant cells or its microenvironment could be integrated into the classic TNM stage classification to help improve risk stratification and patient selection for systemic treatment.

The development of progressive malignant disease certainly encompasses a series of complex and sequential stages. Genetic abnormalities in signaling pathways modify the endogenous machinery of both normal cells and the extracellular matrix (14), and the transformed malignant cells then profit from these endogenous changes to sustain their proliferation through the production of macromolecules, energy, and reactive oxygen species. As a result, the tumor extracellular matrix often undergoes hypoxia and changes in its pH and nutritional status (15). Since HA is one of the main components of the extracellular matrix, it contributes significantly to cell proliferation and migration and may, therefore, be involved in the progression of certain malignant tumors (16). More importantly, an HA coating in solid tumors, through CD44, provides a potential target for hyaluronidase, an enzyme responsible for HA degradation and involved in tumor-responsive drug release (17).

In this scenario, sputum analysis has been used as a non-invasive way to identify altered machinery in the environment of lung cancer cells, including that of NSCLC (18). However, we wanted to investigate whether HA sputum levels in NSCLC patients could be used to detect progressive disease and monitor treatment response compared to a computed tomography scan. Our study used a two-stage design: first, we compared the sputum HA levels in two different concentrations of hypertonic saline solution with the clinicopathological characteristics of patients; then, we compared the sputum HA levels before treatment with the computed tomography scans of tumor responses after CT and CT+IO and outcomes. Our results provided new evidence that NSCLC cells can express matricellular HA in sputum, and that sputum HA level may be a powerful candidate for an *in situ* non-invasive diagnostic tool and can yield a specific machinery phenotype of NSCLC to predict progressive lung disease and treatment response. This is a novel finding by our group, part of which has been previously reported (19).

Since HA is found in the extracellular matrix as either high-molecular-weight (>1000 kDa) or low-molecular-weight HA (<300 kDa), in the current study we tested two different concentrations of hypertonic saline solution (3 and 7%). This design is in agreement with Delpech et al. (20), who found an adequate recovery of HA, with intra- and inter-assay variation coefficients reported as 6 and 12%, respectively. In addition, Garantziotis and colleagues have demonstrated that the biological effects of HA in the regulation of airway injury are dependent on its molecular weight (21). The HA concentration (ng/mg) in 7% saline was significantly related to several factors including tobacco history, tumor stage, phenotype, and mutational status. By examining the sputum, we identified higher HA concentrations in smokers with squamous cell carcinoma and *KRAS* mutation, indicating a possible alteration in this pathway machinery, triggered by the malignant transformation of normal airway cells. It is also worth noting that patients in our study presenting with central lesions had significantly higher HA concentration compared to those suffering from peripheral disease.

Currently, no screening program to detect early-stage lung cancer has been successfully implemented. When compared to chest X-rays in the National Lung cancer Screening Trial (NLST), which included a considerable number of subjects, low-dose computed tomography scan screening resulted in a 20% reduction in mortality (22). However, 7% of patients underwent this invasive diagnostic routine unnecessarily and were free of any signs of lung cancer (23). Therefore, it is crucial to explore novel noninvasive and cost-effective tools that could be routinely used to detect early-stage lung cancer and eventually complement low-dose computed tomography scans. Moreover, it is also worth noting that the analysis of biofluids, such as sputum, might even help to better select patients who can benefit from low-dose computed tomography scans. We found that patients in early-stage disease with sputum HA ≤30.70 ng/mg before treatment achieved a complete tumor response after chemotheraphy and had better progression-free survival (PFS) than those with HA level >30.70 ng/mg. We also found that patients with advanced-stage NSCLC disease who expressed equally low levels of sputum HA before treatment had a complete tumor response and stable disease after CT+IO treatment, with better PFS than those receiving CT alone.

We concluded that, in our cohort of NSCLC patients, sputum HA levels successfully predicted response and aided in treatment monitoring, although with different prognostic implications in early and advanced stages. These data raise the possibility that the concentration of HA levels in the sputum of NSCLC patients may reflect a biochemical reprogramming of the endogenous machinery by malignant cells during progressive disease.
